# Impact of pressure as a tactile stimulus on working memory in healthy participants

**DOI:** 10.1371/journal.pone.0213070

**Published:** 2019-03-14

**Authors:** Mahboobeh Dehghan Nayyeri, Markus Burgmer, Bettina Pfleiderer

**Affiliations:** 1 Medical Faculty and Institute of Clinical Radiology, University Hospital Muenster, Muenster, Germany; 2 Department of Psychosomatic Medicine and Psychotherapy, LVR Clinic, Medical Faculty of the Heinrich-Heine-University Duesseldorf, Duesseldorf, Germany; 3 Department of Psychosomatics and Psychotherapy, University Hospital Muenster, Muenster, Germany; Federal University of Santa Catarina, BRAZIL

## Abstract

Studies on cross-modal interaction have demonstrated attenuated as well as facilitated effects for both neural responses as well as behavioral performance. The goals of this pilot study were to investigate possible cross-modal interactions of tactile stimulation on visual working memory and to identify possible neuronal correlates by using functional magnetic resonance imaging (fMRI). During fMRI, participants (n = 12 females, n = 12 males) performed a verbal n-back task (0-back and 2-back tasks) while tactile pressure to the left thumbnail was delivered. Participants presented significantly lower behavioral performances (increased error rates, and reaction times) during the 2-back task as compared to the 0-back task. Task performance was independent of pressure in both tasks. This means that working memory performance was not impacted by a low salient tactile stimulus. Also in the fMRI data, no significant interactions of *n-back x pressure* were observed. In conclusion, the current study found no influence of tactile pressure on task-related brain activity during n-back (0-back and 2-back) tasks.

## Introduction

The ability to maintain focus on task-relevant information in the presence of interference protects our limited cognitive resources from becoming overloaded. Therefore cognitive control is needed to bridge the gap between the processing of distracting sensory information and goal-directed action [1]. Studies on cross-modal interaction, including vision and tactile sensation [2, 3], have demonstrated inhibitory as well as facilitated effects for both, neural responses in higher sensory association and primary sensory cortices, as well as behavioral performance. Similarly, tactile stimulation of the index finger can induce the perceptual suppression of visual stimuli when tactile and visual information are spatially and temporally consistent [4].

According to the perceptual load hypothesis [5] there are sufficient additional attentional resources available to fully process and identify the distractor in a low cognitive load task. However, in a high-load condition all resources are needed for the processing of the relevant items and therefore no attentional resources remain to process the distractor item.

Previous studies indicated that changes of load in one modality (vision) can affect processing in a different modality (e.g. touch or audition). These cross-modal studies demonstrated that auditory processing was affected negatively during high perceptual load e.g. when participants were asked to detect a tone while performing a visual letter identification task [6] or in another experiment to ignore the auditory input [7]. However, there were studies reporting findings which were in contrast to those results [8–10]. It was also shown that tactile spatial attention can alter visual event-related potentials (ERPs) [11]. Zimmer et al. showed that higher cross-modal activity in the visual cortex contralateral to the position of spatially congruent visuo-tactile stimuli was independent of the level of load [12]. Spence & Driver (2004) also demonstrated cross-modal congruency effects of spatial attention by investigating responses to visual, auditory, and tactile stimuli [13]. The association of activities in areas related to the multimodal attentional system with brain activity in areas representing common regions of space for different modalities suggests a link between spatial attention and cross-modal integration [14]. By selectively manipulating the relation of various sensory inputs it is possible to investigate by fMRI whether a brain region responds to a specific cross-modal relation or whether co-stimulation deviates from uni-sensory stimulation [2].

Control of attention involves coordinated activity of parietal and prefrontal brain regions. These regions sustain attention for processing and filtering relevant information in the presence of irrelevant information [15]. Two attention networks have been described: a bilateral dorsal executive attention system (lateral prefrontal and parietal) and a more right-lateralized ventral affective attention (ventral fronto-parietal) network, involved in responding to salient events [16, 17].

Another brain network known to play a role in attention is the salience network (SN). Its key nodes are the anterior insula, dorsal anterior cingulate cortex (dACC) and other subcortical and limbic structures including the amygdala. The SN is involved in the detection of and responding to transient behaviorally salient stimuli [18]. It plays an important role in attentional control [19] and mediates switching between activation of the lateral fronto-parietal central executive network (CEN) and the medial fronto-parietal default mode network (DMN). It has been shown that task-irrelevant salient distractors that attract attention did not activate the ventral system [20], despite activating the dorsal system.

Tactile pressure has been a relatively unattended sensory modality in cognitive research. Previous tactile perception studies have investigated the existence of cross-modal attentional interaction of the sensory modalities such as auditory, visual, and tactile stimuli [21, 22].

It has been demonstrated that high working memory load interfered with the ability to perform a vibro-tactile selective attention task [23]. However one question that remains to be answered is whether tactile non painful pressure as a low salient sensory stimulus also interferes with working memory processing in healthy participant. If this is the case, neurobiological substrates underlying this interference need to be identified. To answer these questions in this study, healthy participants performed an n-back task while tactile pressure was administered. Simultaneously, their brain activity was assessed by functional magnetic resonance imaging (fMRI). fMRI is an established neuroimaging method [24] to investigate cerebral functions in different paradigms.

The main aim of this study was to investigate cross-modal interactions of visual n-back working memory on tactile stimuli by fMRI to identify the cortical regions involved with a special focus on somatosensory cortical responses. Brain activities of participants were assessed while performing a visual n-back task with 2 different memory loads (0-back and 2-back) with a simultaneous tactile stimulation of the thumbnail.

The following hypotheses were tested:

- The tactile pressure stimuli of low salience affect working memory capacity and processing negatively.
- Alterations in brain activity caused by pressure are expected to occur mainly in core regions of the ventral attention network playing a role in the bottom-up control of attention to salient stimuli.

## Materials and methods

### Participants

The study sample (N = 24) consisted of 12 male and 12 female healthy right-handed participants (23 ± 2.69 [mean age ± SD]; range 19–28 years) with normal or corrected-to-normal vision and no history of neurological or major psychiatric disorders like psychosis or substance abuse. All participants were German native students at the University of Muenster, Germany. To control for possible effects of psychopathology on the results and to rule out an effect of handedness, participants completed several questionnaires before fMRI data acquisition. These included the Edinburgh Handedness Inventory to assess handedness [25], the German version of the Patient Health Questionnaire (PHQ)-9 [26], a standardized screening tool for depression and the German version of the perceived stress scale (PSS) 10 [27], a standardized instrument to measure perceived stress. All participants were classified as right-handed and none of the participants had to be excluded based on these tests. Demographical and clinical characteristics of the participants are reported in Table 1.

**Table 1:** Demographical and clinical characteristics of the participants.

	Mean ± SD
Age (years)	23.8 ± 2.69
Sex (females, males)	12F, 12M
Education (years)	15 ± 2.64
Handedness: EHI	83.5 ± 14.3%*
PHQ-9 (depression)	4.04± 3.65
PSS 10 (stress)	9.50 ± 6.14

SD: standard deviation, EHI: Edinburgh Handedness Inventory, PHQ-9: Patient Health Questionnaire for depression, PSS 10: perceived stress scale 10 (normal range up to 14).

*All participants were classified as being right-handed.

All participants received a 20 € compensation for their participation. Prior to participation, participants were informed about the study protocol in spoken and written form and gave their written informed consent. The study was approved by the ethics committee of the Medical Faculty of the University Muenster and the Westphalian Chamber of Physicians in Muenster (2011-030-f-S).

### Experimental paradigm

The n-back paradigm was used in this study to evoke graded memory load of the relevant task [28]. In this n-back paradigm, participants are required to monitor a series of quickly changing letters. They were instructed to press the left button of a response box with their right index finger whenever a target letter appeared on the screen and to press the right button with the right middle finger if no target letter was seen. In the baseline 0-back task participants were asked to respond to the letter X as target, in the 2-back task participants had to decide whether the letter shown on the display was the same as the letter presented two trials earlier [29]. The 0-back condition is a sensory-motor control condition that requires sustained attention without working memory demand [30]. The 2-back condition represents a memory task with high cognitive load.

An MR compatible stimulation device was used to apply pressure to the left thumbnail. It consisted of a plastic piston with a surface area of 1 cm^2^ that can apply pressure up to a maximum of 8 kg with a pneumatic system (in-house development). Prior to the experiment inside the MR-scanner a pressure of 1 kg/cm^2^ (~98 kPascal) was applied to the left thumbnail. Participants were asked to judge whether the tactile stimulus was perceived as pressure without experiencing any pain. None of the participants reported any pain. Therefore, a 1 kg/ cm^2^ pressure was subsequently used for all participants during the MRI scanning as the tactile non painful pressure.

**Fig 1:**
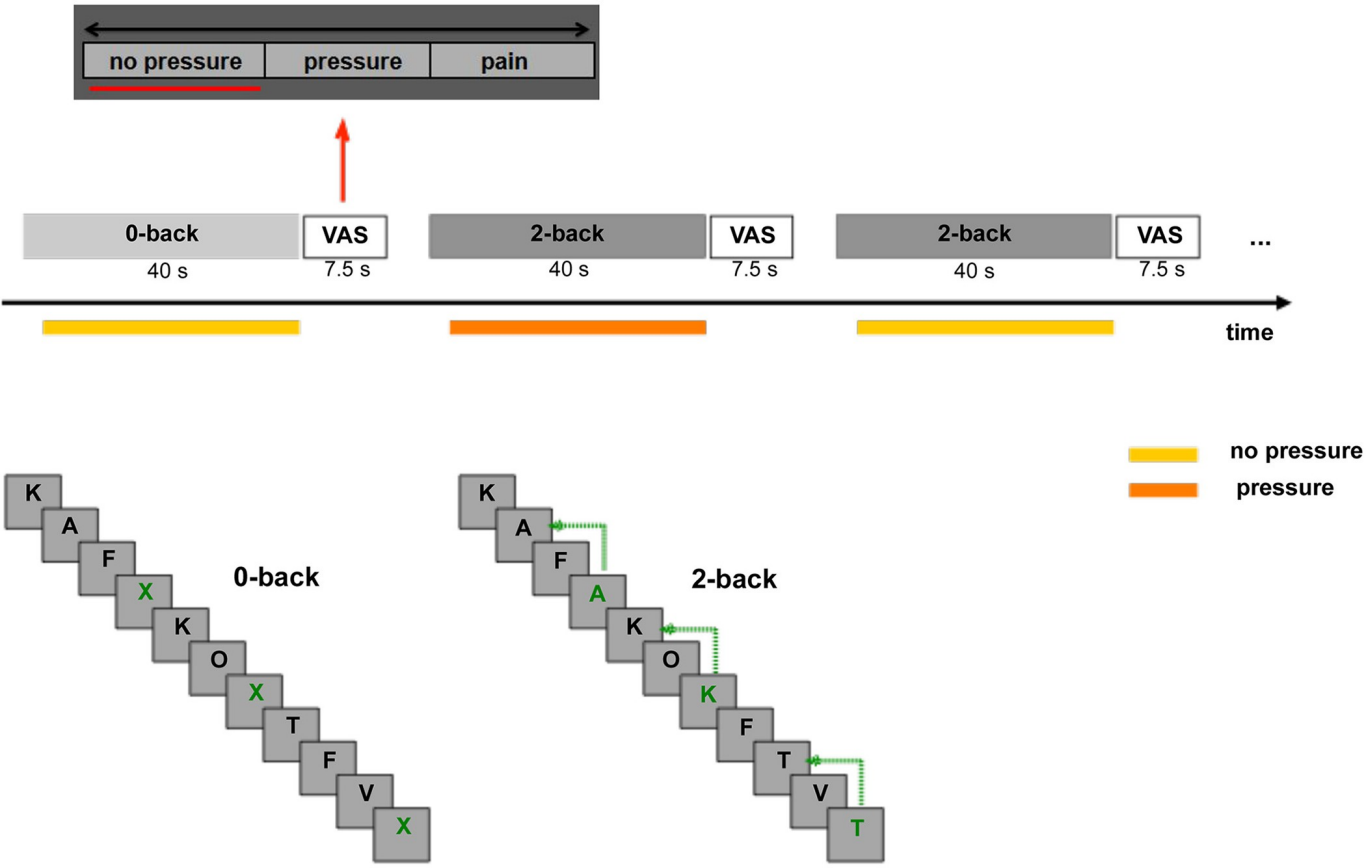
Schematic illustration of the used n-back paradigm with 0-back and 2-back tasks, VAS: visual analog scale with three items (no pressure, pressure and pain). The grey rectangles each depict one task block. The thick lines represent the pressure (dark) and without the pressure (light) block condition.

The experiment started outside the scanner with a practice block of n-back tasks without any concurrent pressure stimuli. The experiment inside the scanner consisted of one run including 24 blocks; each lasting 40 seconds (s) (Fig 1). Four experimental conditions as combinations of the memory and sensory tasks (2-back with pressure, 2-back without pressure, 0-back with pressure and 0-back without pressure) were presented six times each in a pre-randomized order. Each block started with a brief (5 s) visual information informing the participants whether the 0-back or 2-back condition was about to follow. The instruction was followed by the 16 letter stimuli each presented for 500 ms followed by 2000 ms ± jitter (range: 0–900 ms) of a gray mask to sustain the participants’ attention and to prevent fatigue and habituation. The ratio of target to non-target letters presented per block was 32%, with a total of 30 target stimuli for each condition.

During the “pressure” blocks participants received continuous pressure across the entire block except for the first letter. During “no pressure” blocks no pressure was applied. This resulted in a total of 12 “pressure” blocks (6 of 0-back task, 6 of 2-back task) and 12 “no pressure” blocks, using the restriction to not present more than 2 pressure blocks consecutively to minimize the effects of expectancy and habituation.

Pressure intensity ratings were made after each block with a visual analog rating scale (VAS) with three items (no pressure, pressure, and pain) shown on the screen (7.5 s) in which the subjects moved the bar to the appropriate point on the scale using a button box (Fig 1). All visual stimuli in current study were delivered using the Presentation Software (Version 16.5, www.neurobs.com). Reaction time to letters, total numbers of correct responses, and errors were recorded.

### Image acquisition

The fMRI experiment was conducted in a 3 T scanner (Magnetom Prisma^fit^, Siemens Medical Systems, Erlangen, Germany) using a standard 20-channel head coil. T_2_*-weighted functional images were acquired using a standard single-shot, gradient echo-planar imaging (EPI) pulse sequence sensitive to blood oxygenation level dependent (BOLD) contrast with imaging parameters of repetition time (TR) = 2500 ms, echo time (TE) = 30 ms, flip angle = 90°, field of view (FOV) = 220 mm^2^ with a 74 × 74 data acquisition matrix. Each volume consisted of forty five adjacent axial slices with a slice thickness of 2.7 mm and 11% gap, resulting in a voxel size of 3×3×3 mm^3^. Images were acquired in interleaved order -25° angulated the AC–PC (anterior and posterior commissures) plane in order to capture the whole brain. A run consisted of 549 successive brain volumes. Parallel acquisition (SENSE, acceleration factor = 2) was used, trading gray-white contrast for a reduced acquisition time.

A high-resolution T_1_-weighted anatomical image was additionally acquired using a standard Siemens 3D magnetization-prepared gradient echo (MPRAGE) sequence with cubic voxels of 1 mm^3^ edge length, TR = 2130 ms, TE = 2.28 ms, inversion time = 900 ms, flip angle = 8° degrees, FOV = 256 mm^2^, 256 × 256 matrix and 192 slices, parallel imaging with GRAPPA (parallel imaging factor = 2). MPRAGE scans were acquired for each participant directly after functional imaging for the purpose of co-registration during image preprocessing.

### Data analysis

#### Behavioral data and performance of the n-back task

The percentage of errors was calculated as a measure of error rates (ERs). The mean reaction times (RTs) were used as a measure of response speed (excluding RTs of the first response of each block for the first letter, incorrect responses, anticipated responses (RT<150 ms), and missed responses). The RTs and the ERs were compared using a repeated measure 2-factorial analysis of variance (ANOVA) with *memory load* (2-back vs. 0-back) and *pressure* (with vs. without tactile stimulation) as within-subjects factors.

All statistical analyses investigating behavioral measures were assessed using Predictive Analytics Software SPSS (IBM Corp. Released 2013. IBM SPSS Statistics for Windows, Version 22.0. Armonk, NY: IBM Corp.).

#### Preprocessing and statistical analysis of fMRI data

A total of 549 volumes were obtained for the fMRI run. The first five volumes were discarded from the data set and not analyzed in order to avoid equilibration effects. FMRI data was preprocessed and analyzed using Statistical Parametric Mapping software packages (SPM 8; Wellcome Centre for Human Neuroimaging, London, UK) running under Matlab 10 (Mathworks, Sherborn, MA, USA). Preprocessing steps included realignment (motion correction) with the rigid-body transformation matrices (realignment to the first volume), slice-timing correction, co-registration to a structural T1 image, spatial normalization to the Montreal Neurological Institute (MNI) template (standard EPI template) and T1 generating 2 x 2 x 2 mm resolution images and smoothing with an isotropic Gaussian kernel of 8 mm^3^ full width half maximum (FWHM). The six realignment parameters obtained during preprocessing were selected as first-level covariates to control for movement-related artifacts.

The general linear model (GLM) in SPM 8 was used to perform a statistical analysis of the BOLD signals with a canonical hemodynamic response function as well as its time and dispersion derivatives. A 128 s high-pass frequency filter removed low-frequency drifts and physiological artifacts in the BOLD signals. A first-level model was implemented for each participant by compiling all blocks for each condition respectively. For each individual, four contrasts: 0-back with and without tactile stimulation and 2-back with and without tactile stimulation were calculated and submitted to a second-level random effects ANOVA to test for significant differences in brain activity. The following factors were analyzed by a 2-factorial ANOVA in the ‘full factorial’ design option in SPM 8: the dependent within-subject factors tactile stimulation (with and without tactile pressure) and memory demand (2-back and 0-back) resulting in a 2x2 design. According to our hypotheses the main effects of memory demand (*n-back*), tactile stimulation (*pressure*) as well as the interactions between the factors (*pressure* x *n-back*) were assessed. Significant main effects and interaction were further analyzed by differential post-hoc analyses.

Anatomical localization of activated brain regions for all analyses was determined by reference to the standard stereotaxic atlas by MNI and labeled following the nomenclature of the Automated Anatomical Labeling (AAL) atlas [31].

In this pilot study, all statistical maps were reported at voxel-level (p < 0.05, family wise error (FWE) [32] corrected for multiple voxel comparisons and using a spatial extend threshold of 20 voxels. For illustration, the significant clusters were overlaid on a standardized anatomical MNI-normalized template (Colin_27_T1).

## Results

### Behavioral results

To assess whether the tactile stimulus was sufficiently salient to be perceived, participants were asked to rate the tactile stimulation after each block. The analysis of the ratings showed that participants were able to distinguish 90% of the tactile stimuli to either the pressure or non-pressure condition correctly. There was no significant effect of *n-back* (F (1,23) = 2.772, p = 0.110, partial Eta^2^ = 0.108) and no significant effect of *pressure* (F (1,23) = 0.000, p = 0.999, partial Eta^2^ = 0.000) as well as no significant interaction of *n-back x pressure* (F (1,23) = 0.271, p = 0.608, partial Eta^2^ = 0.012) on the rating of tactile stimuli. Results are summarized in Table 2.

**Table 2:** Correct assignment of stimuli to either the presence of pressure or non-pressure (mean in %) for the different conditions. The analysis of the responses showed that almost over 90% of the tactile stimuli were assigned correctly (N = 24).

condition	mean (%)	SD
2-back, pressure	93.06	19.61
2-back, no pressure	92.36	21.41
0-back, pressure	94.46	12.66
0-back, no pressure	95.14	20.55

SD = standard deviation

We found a significant main effect of memory load on reaction times (F (1,23) = 109.225, p< 0.001, partial Eta^2^ = 0.826): participants were significantly slower in their response under high memory load (2-back) than under no memory load (0-back). Similarly, there was also a significant main effect of memory load on error rates (ERs) (F (1,23) = 8.45, p = 0.008, partial Eta^2^ = 0.269). ER was significantly lower under no load than under high load (2-back). There was no significant main effect of tactile stimulation on RT (F (1,23) = 0.125, p = 0.727, partial Eta^2^ = 0.005) or ER (F (1,23) = 0.014, p = 0.905, partial Eta^2^ = 0.001) (Table 3).

**Table 3:** Mean error rates (ER) (%) and mean reaction times (RT) (ms) for different conditions. Participants were significantly slower in their response (RT) under 2-back than under 0-back. Similarly, ER was significantly lower under 0-back than under 2-back. There was no significant difference of tactile stimulation by pressure on RT or ER (N = 24).

condition	RT		ER	
	mean	SD	mean	SD
2-back, pressure	867.68	200.28	11.76	17.56
2-back, no pressure	866.41	203.30	11.02	13.64
0-back, pressure	650.64	127.60	1.94	1.83
0-back, no pressure	646.68	142.49	2.50	3.41

SD = standard deviation

There was no significant interaction of *n-back x pressure* for RT (F (1,23) = 0.110, p = 0.743, partial Eta^2^ = 0.005) or for ER (F (1,23) = 1.084, p = 0.309, partial Eta^2^ = 0.045); tactile pressure did not interfere with the participants’ performance during the n-back tasks. Corresponding ERs and RTs are summarized in Table 3.

### fMRI results

**Fig 2:**
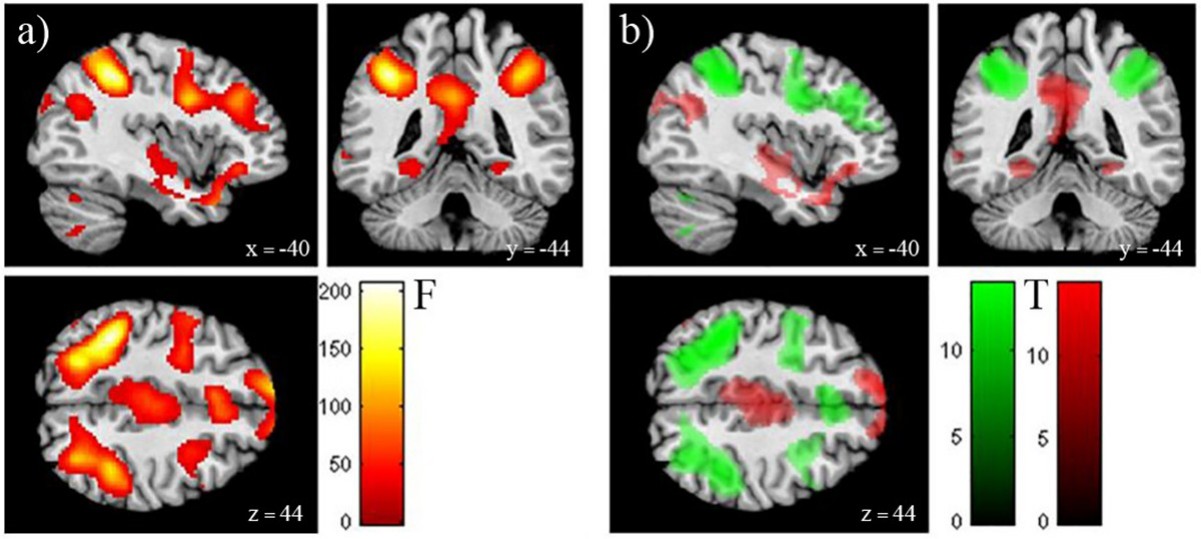
BOLD activity map of main effects and post-hoc analyses of a 2-factorial ANOVA with tactile stimulation and memory demand as factors. The significant clusters for the main effect of *n-back* (a) and related differential post-hoc contrasts *0-back >2-back* (red) and 2*-back> 0-back* tasks (green) (b) are shown. a) A main effect of *n-back* was observed for regions including the left superior medial, right middle frontal gyrus, as well as bilateral inferior parietal and middle temporal gyrus, left posterior cingulum, insula, occipital lobe and cerebellum bilaterally. b) Green regions are related to an increase in activation with increased task difficulty (*2-back>0-back*) within a fronto-parietal executive network. Red regions depict areas that activate with reduced task difficulty (*0-back>2-back*) within the default mode network (DMN). Activations were thresholded at a whole-brain FWE corrected p< 0.05 with an extent threshold of 20 voxels.

Main effects of *n-back* and *pressure* (Table 4) were observed. The post-hoc analysis revealed that the *n-back* main effect was related to a higher activity during the 2-back task compared to the 0-back (contrast: *2-back>0-back*) with activities in the left inferior parietal lobule (Brodmann Area (BA) 39/40), right middle frontal gyrus (dorsolateral prefrontal cortex (DLPFC) (BA9/46)), insula and cerebellum bilaterally (Table 5, Fig 2).

**Table 4:** F-contrast for the main effect of *pressure* and *n-back*. Activations were thresholded at a whole-brain FWE corrected p< 0.05 with an extent threshold of 20 voxels. The MNI Coordinates are the same as peak voxel locations.

Contrast	Brain Region	BA	MNI Coordinates (mm)	k	F-value	p_FWE-corr._
			x y z			
Main effect of ‘*Pressure*’	Right rolandic operculum		52–20 23	168	38.4242	0.000
	Right postcentral gyrus	1	54–16 48	33	31.0202	0.006
Main effect of ‘*n-back*’	Left medial superior frontal gyrus	10	-4 60 14	5833	212.86	0.000
	Left inferior parietal gyrus	40	-40–44 44	3242	197.75	0.000
	Left middle temporal gyrus	20	-62–12–20	3845	187.35	0.000
	Left posterior cingulum	31	-2–50 28	3329	164.11	0.000
	Right middle frontal gyrus	6	32 4 60	6894	145.46	0.000
	Right inferior parietal gyrus	40	40–44 44	2969	119.60	0.000
	Left angular gyrus	39	-50–64 26	1163	104.51	0.000
	Right middle temporal gyrus	21	62–6–18	2482	88.1212	0.000
	Right cerebellum(Pyramis)		30–82–34	330	86.5050	0.000
	Right insula		32 22 0	207	66.7777	0.000
	Left insula (anterior insula)		-28 24 0	158	54.5353	0.000
	Right cerebellum (Tuber)		38–66–28	193	48.5151	0.000
	Left cerebellum (Pyramis)		-8–74–24	247	46.1414	0.000
	Right angular gyrus	39	56–64 30	88	45.5454	0.000
	Right inferior parietal gyrus, supramarginal gyrus	40	66–30 30	286	43.5252	0.000
	Left cerebellum (Pyramis)		-32–82–34	84	39.2828	0.000
	Left cerebellum (Tuber)		-34–66–28	71	37.5252	0.001
	Left superior occipital gyrus	19	-20–96 22	48	32.8282	0.003
	Left middle temporal gyrus	21	-64–48–4	39	32.5959	0.003

xyz = coordinates of the standard MNI brain implemented in SPM8; k = cluster-size of contiguous voxels, BA = Brodmann area, MNI = Montreal Neurological Institute, p-values correspond to peak voxels of the clusters.

**Table 5:** Post-hoc tests to analyze the main effect of *n-back*. Activations from significant clusters for contrast *2-back>0-back* and *0-back>2-back* (t-contrast). Activations were thresholded at a whole-brain FWE corrected p < 0.05 with an extent threshold of 20 voxels. The MNI Coordinates are the same as peak voxel locations.

Contrast	Brain Region	BA	MNI Coordinates (mm)	k	T- value	P_FWE-corr._
			x y z			
*2-back>0-back*	Left inferior parietal gyrus	40	-40–44 44	6470	14.06	0.000
	Right middle frontal gyrus	6	32 4 60	7448	12.06	0.000
	Right insula (anterior insula)		32 22 0	228	8.1717	0.000
	Left insula (anterior insula)		-28 24 0	183	7.3838	0.000
	Right cerebellum (Tuber)		38–66–28	230	6.9696	0.000
	Left cerebellum (Pyramis)		-8–74–24	313	6.7979	0.000
	Left cerebellum (Tuber)		-34–66–28	102	6.1313	0.000
*0-back>2-back*	Left medial superior frontal gyrus	10	-4 60 14	6164	14.59	0.000
	Left posterior cingulum	31	-2–50 28	3603	12.81	0.000
	Left angular gyrus	39	-50–64 30	1240	10.22	0.000
	Right middle temporal gyrus	21	62–6–18	2881	9.39	0.000
	Right cerebellum (Pyramis)		30–82–34	347	9.3030	0.000
	Right Angular Gyrus	39	56–64 30	113	6.7575	0.000
	Right supramarginal gyrus	40	66–30 30	373	6.6060	0.000
	Right superior occipital gyrus	19	28–92 20	105	6.2828	0.000
	Left cerebellum (Pyramis)		-32–82–34	106	6.2727	0.000
	Left middle occipital gyrus	18	-20–96 22	73	5.7373	0.002
	Left middle temporal gyrus	21	-64–48–4	69	5.7171	0.002
	Right precentral gyrus	6	44–12 60	41	5.3333	0.007

xyz = coordinates of the standard MNI brain implemented in SPM8; k = cluster-size of contiguous voxels, BA = Brodmann area, MNI = Montreal Neurological Institute, p-values correspond to peak voxels of the clusters.

These regions are related to an increase in activation with increased task difficulty (*2-back>0-back*) within a fronto-parietal executive and salience networks. Areas that decrease their activity with task difficulty (*0-back>2-back*) include the default mode network (DMN).

**Fig 3:**
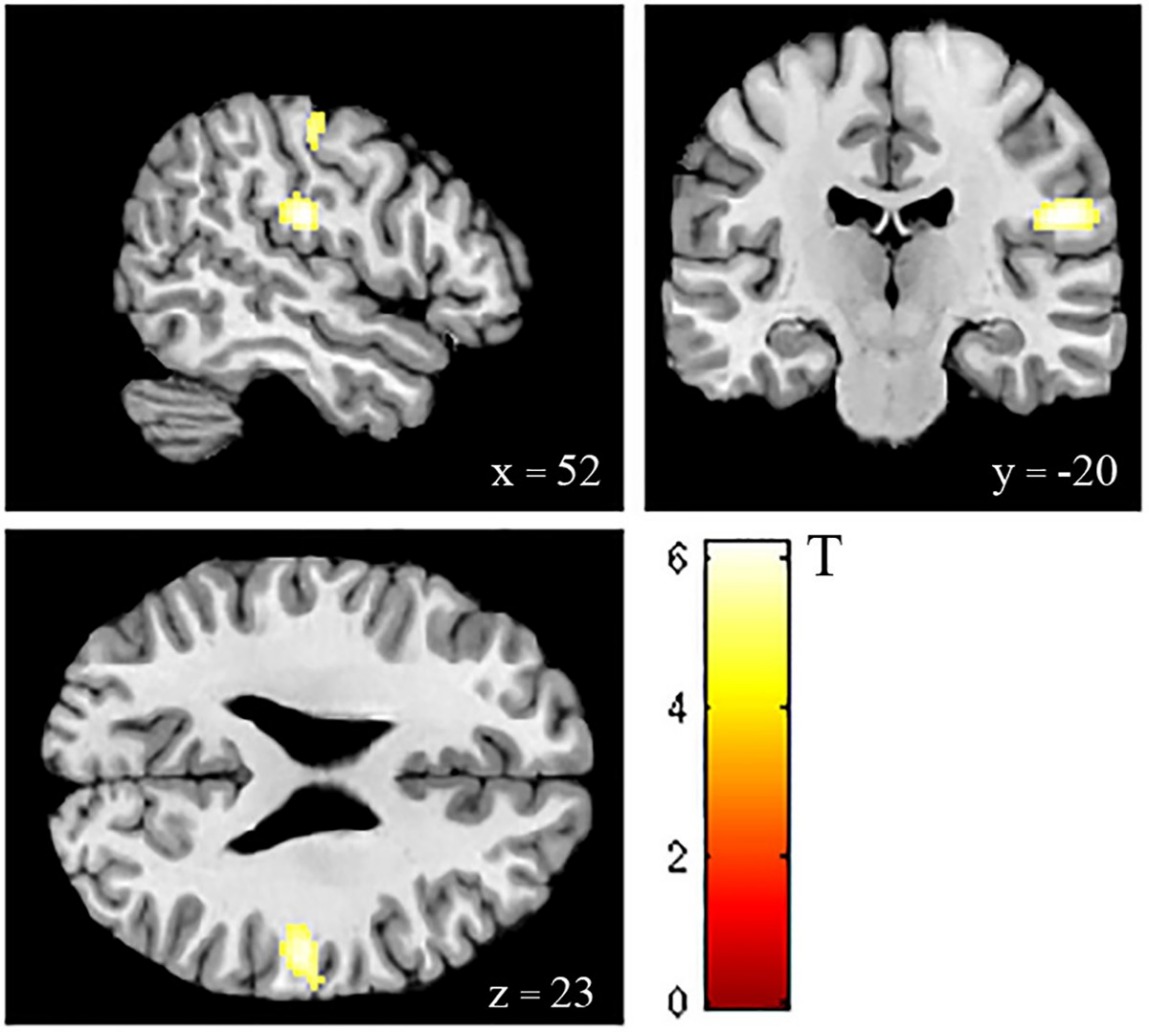
Bold activity map depicting the post-hoc analysis (contrast *pressure > no pressure)* to explain the significant main effect of pressure in an ANOVA (2x2) with tactile pressure and memory demand as factors. This analysis showed significant increased activity in the contralateral postcentral gyrus and the rolandic operculum during tactile stimulation in contrast without stimulation. Activations were thresholded at a whole-brain FWE corrected p< 0.05 with an extent threshold of 20 voxels.

The significant main effect of *pressure* can be explained by increased activity of the contralateral postcentral gyrus and the rolandic operculum during tactile stimulation in contrast to blocks without stimulation (contrast: *pressure>no pressure*) (Table 6, Fig 3). The interaction *n-back x pressure* was not significant.

**Table 6:** Post-hoc tests to assess the main effect of *pressure*. Activations from significant clusters for contrast pressure> no pressure (t-contrast), activations were thresholded at a whole-brain FWE corrected p< 0.05 with an extent threshold of 20 voxels. The reverse contrast “no pressure > pressure” yielded no significant results. The MNI Coordinates are the same as peak voxel locations.

Contrast	Brain Region	BA	MNI Coordinates (mm)	k	T-value	p_FWE-corr._
			x y z			
*Pressure>no pressure*	Right rolandic operculum		52–20 23	213	6.2020	0.000
	Right postcentral gyrus	1	54–16 48	52	5.5757	0.003

xyz = coordinates of the standard MNI brain implemented in SPM8; k = cluster-size of contiguous voxels, BA = Brodmann area, MNI = Montreal Neurological Institute, p-values correspond to peak voxels of the clusters.

## Discussion

The goal of this fMRI study was to investigate the possible influence of low salient tactile stimuli (pressure) on performance and related brain activity in a working memory task. To do so, healthy participants were asked to perform a visual n-back paradigm (0-back, 2-back) combined with pressure as a sensory stimulus.

### Behavioral results

In line with the results of previous studies [30], the high load task (2-back) was significantly more cognitively demanding than the 0-back task: Participants responded significantly slower and made more errors in the 2-back compared to the 0-back task. The 0-back condition is a sensory-motor control condition that requires sustained attention, but no working memory demand (no memory load) [30]. The error rates and reaction times of our participants were comparable to other studies [30, 33, 34]. Tactile stimulation was sufficiently salient in our participants. Over 90% of the stimuli were assigned correctly to the presence of pressure or non-pressure (Table 2).

The behavioral analyses of reaction times and error rates in the n-back task showed that these tactile stimuli of low salience did not affect working memory performance. Performance of the n-back task was comparable with and without tactile stimulation under high as well as under no memory load (0-back) (Table 3). It may therefore be feasible to assume that the tactile stimulation was salient in participants but did not compete with cognitive processing. There are sufficient attentional resources available to identify and fully process the n-back task despite tactile stimulation.

### fMRI results

The BOLD findings confirmed previous results of brain activity for verbal n-back tasks [35, 36] as well as for processing of tactile stimuli [37–40]. Brain activity was in line with previous literature (Tables 3–5, Figs 2 and 3).

Our fMRI results revealed fronto-parietal and subcortical neural activation patterns corresponding to working memory load [41]. This was supplemented by significantly higher error rates and reaction times with 2-back task, which confirmed the successful manipulation of n-back task. The fMRI activations of the fronto-parietal network corroborated previous findings of brain activity when using this visual working memory task [36, 41, 42].

Our results were also in line with studies reporting increased activation in regions that are responsible for maintaining and processing verbal information in healthy participants. The higher activity of the middle frontal gyrus (MFG) may reflect higher cognitive demands when performing the 2-back task, it could also reflect stronger top-down mediated effort. The 2-back task may increase attention and engage working memory, thus leading to greater activity of the MFG, as reported in our study. The DLPFC as a part of MFG is associated with cognitive control in relation to top-down goals and bottom-up sensory stimulation [1, 43]. The activity of right MFG has been shown to be correlated with the activity of both attention networks [44] and has been reported as a region for linking the dorsal with the ventral attention network [45]. Participants displayed greater activity in the inferior parietal gyrus that is hypothesized to play an important role in maintaining attention and responding to salient stimuli [46, 47].

Our results corroborated previous findings that the postcentral gyrus and the rolandic operculum are involved in brain activity evoked by tactile stimulation [38, 48–50]. The human somatosensory system includes the primary (SI) and the secondary somatosensory cortices (SII), with SI is located in the postcentral gyrus in the anterior parietal lobe. Previous results suggested the involvement of SI in more perceptual aspects of tactile stimulus recognition [51].

The mechanical stimulation on the left thumbnail resulted in activation of the right postcentral gyrus. This was opposite to the stimulation site and consistent with the data on tactile thumb stimulation. The thumb mapping is located more laterally in S1 [52]. This finding is also consistent with previous human neuroimaging research on the somatotopic location of the hand within SII [53–55]. Functional neuroimaging studies reported activation in postcentral gyrus (presumably SI), parietal operculum (presumably SII) in response to vibro-tactile stimulation on the hand [48].

Some studies have reported enhanced activity in SI when tactile stimulus is made relevant to performance of a task [56, 57], but other studies have not found a task-relevant modulation within SI [49, 58]. The findings of task-relevant and cross-modal modulation of SI suggest that both relevance and modality of stimuli can affect the excitability of the sensory cortex. Furthermore, some studies have provided evidence that both SI and SII are modulated by tactile spatial attention processing [59, 60]. However, another study described no attention effect for SI, whereas SII seemed to play an important role in somatosensory attention irrespective of stimulus characteristics [61]. Results from previous studies suggested that SII is involved in tactile processing [38, 49, 50] and that higher level of tactile processing enhances activation in the parietal cortex including the somatosensory cortex [62, 63].

Another aim of this study was to assess whether the presence of tactile pressure impacts the processing and related brain activity in a working memory task and how higher visual working memory load will impact the tactile task. We did not observe any interaction of *n-back x pressure*, indicating that the load seems to have no influence on cross-modal processes. This is in line with several studies also showing no influence of various loads on cross-modal processing and multisensory integration [10, 12]. This finding can also be explained by the ‘automaticity theory’ [64, 65], i.e. the assumption of automatic cognitive processing without requiring attention resulting in insensitivity to load manipulations in the secondary task processing [8, 66].

## Limitations and future directions

The study sample of 24 participants with 12 female and 12 male young medical students is rather small and homogenous. Consequently, this study needs to be considered a pilot study to investigate potential differences in brain activity in response to tactile stimulation during a visual working memory task. The number of participants might have been too small to measure subtle differences.

In contrast to our assumptions and in line with the behavioral results we could not observe any above-threshold clusters for the interaction of *n-back x pressure*. One might argue that the tactile salience in our paradigm was not sufficient to compete with the task demands. However as the participants were able to assign 90% of the tactile stimuli to either the pressure or non-pressure category correctly, is it feasible to assume that the tactile stimulus was above threshold-level. Therefore, the absence of absent interaction with *pressure x n-back* cannot be explained by low salience of the pressure stimulus. However, using constant tactile pressure stimulation in a block design is of risk for a fast adaptation to this stimulus [67]. In this way, the onset of tactile pressure occurrence can be detected in order to report a pressure block, but will not remain an effective distractor during the course of the visual n-back stimulus sequence, with likely effects to both, behavioral performance and brain activity. Therefore, the negative results could also be caused by the experimental limitations due to a possible fast reduction of the tactile salience due to sensory adaptation during the course of a single pressure block [67]. Possible experimental improvements for further directions could be providing randomized tactile pressure trials instead of pressure blocks and analyzing the data according to the expected tactile pressure adaptation (i.e. considering only the first trials after pressure onset).

It may also be argued that in our group of participants the 2-back task was not difficult enough to fully harness the shared resources in the brain. This cannot be ruled out completely, even though our behavioral results clearly showed differences between 0-back and 2-back pointing to a higher memory load of the 2-back task. However, a more difficult cognitive task like the 3-back task, which was not included in the present study, might be able to reveal different effects of distractors by requiring higher cognitive loads.

## References

[1] Miller BT, D'Esposito M. Searching for "the top" in top-down control. Neuron. 2005 11 23;48(4):535–8. 10.1016/j.neuron.2005.11.002 16301170

[2] Driver J, Noesselt T. Multisensory interplay reveals crossmodal influences on 'sensory-specific' brain regions, neural responses, and judgments. Neuron. 2008 1 10;57(1):11–23. 10.1016/j.neuron.2007.12.013 18184561 PMC2427054

[3] Calvert GA. Crossmodal processing in the human brain: insights from functional neuroimaging studies. Cereb Cortex. 2001 12;11(12):1110–23. 11709482 10.1093/cercor/11.12.1110

[4] Ide M, Hidaka S. Tactile stimulation can suppress visual perception. Sci Rep. 2013 12 13;3:3453 10.1038/srep03453 24336391 PMC3861798

[5] Lavie N. Perceptual load as a necessary condition for selective attention. J Exp Psychol Hum Percept Perform. 1995 6;21(3):451–68. 7790827 10.1037//0096-1523.21.3.451

[6] Raveh D, Lavie N. Load-induced inattentional deafness. Atten Percept Psychophys. 2015 2;77(2):483–92. 10.3758/s13414-014-0776-2 25287617 PMC4677383

[7] Regenbogen C, De Vos M, Debener S, Turetsky BI, Mossnang C, Finkelmeyer A, et al Auditory processing under cross-modal visual load investigated with simultaneous EEG-fMRI. PLoS One. 2012;7(12):e52267 10.1371/journal.pone.0052267 23251704 PMC3522643

[8] Otten LJ, Alain C, Picton TW. Effects of visual attentional load on auditory processing. Neuroreport. 2000 3 20;11(4):875–80. 10757537 10.1097/00001756-200003200-00043

[9] Zhang P, Chen X, Yuan P, Zhang D, He S. The effect of visuospatial attentional load on the processing of irrelevant acoustic distractors. Neuroimage. 2006 11 1;33(2):715–24. 10.1016/j.neuroimage.2006.07.015 16956775

[10] Haroush K, Hochstein S, Deouell LY. Momentary fluctuations in allocation of attention: cross-modal effects of visual task load on auditory discrimination. J Cogn Neurosci. 2010 7;22(7):1440–51. 10.1162/jocn.2009.21284 19580389

[11] Eimer M, Driver J. An event-related brain potential study of cross-modal links in spatial attention between vision and touch. Psychophysiology. 2000 9;37(5):697–705. 11037046

[12] Zimmer U, Macaluso E. Processing of multisensory spatial congruency can be dissociated from working memory and visuo-spatial attention. Eur J Neurosci. 2007 9;26(6):1681–91. 10.1111/j.1460-9568.2007.05784.x 17880400

[13] Spence C, Pavani F, Driver J. Spatial constraints on visual-tactile cross-modal distractor congruency effects. Cogn Affect Behav Neurosci. 2004 6;4(2):148–69. 15460922 10.3758/cabn.4.2.148

[14] Macaluso E, Driver J. Spatial attention and crossmodal interactions between vision and touch. Neuropsychologia. 2001;39(12):1304–16. 11566313 10.1016/s0028-3932(01)00119-1

[15] Corbetta M., Shulman G.L. Control of goal-directed and stimulus-driven attention in the brain. Nat Rev Neurosci. 2002;3(3):201–15. 10.1038/nrn755 11994752

[16] Fox MD, Corbetta M, Snyder AZ, Vincent JL, Raichle ME. Spontaneous neuronal activity distinguishes human dorsal and ventral attention systems. Proc Natl Acad Sci U S A. 2006;103(26):10046–51. 10.1073/pnas.0604187103 16788060 PMC1480402

[17] Downar J., Crawley A.P., Mikulis D.J., Davis K.D. A cortical network sensitive to stimulus salience in a neutral behavioral context across multiple sensory modalities. J Neurophysiol. 2002;87(1):615–20. 10.1152/jn.00636.2001 11784775

[18] Seeley WW, Menon V, Schatzberg AF, Keller J, Glover GH, Kenna H, et al Dissociable intrinsic connectivity networks for salience processing and executive control. J Neurosci. 2007 2 28;27(9):2349–56. 10.1523/JNEUROSCI.5587-06.2007 17329432 PMC2680293

[19] Crottaz-Herbette S, Menon V. Where and when the anterior cingulate cortex modulates attentional response: Combined fMRI and ERP evidence. J Cogn Neurosci. 2006;18(5):766–80. 10.1162/jocn.2006.18.5.766 16768376

[20] de Fockert J, Rees G, Frith C, Lavie N. Neural correlates of attentional capture in visual search. J Cogn Neurosci. 2004 6;16(5):751–9. 10.1162/089892904970762 15200703

[21] Caclin A, Soto-Faraco S, Kingstone A, Spence C. Tactile "capture" of audition. Percept Psychophys. 2002 5;64(4):616–30. 12132762 10.3758/bf03194730

[22] Spence C. Crossmodal spatial attention [Internet]; 2010 [cited 23 July 2015].

[23] Dalton P, Lavie N, Spence C. The role of working memory in tactile selective attention. Q J Exp Psychol (Hove). 2009 4;62(4):635–44. 19096988 10.1080/17470210802483503

[24] Huettel SA, Song AW, McCarthy G. Functional magnetic resonance imaging Third ed Sunderland, Massachusetts, U.S.A.: Sinauer Associates, Inc., Publishers; 2014.

[25] Oldfield RC. The assessment and analysis of handedness: the Edinburgh inventory. Neuropsychologia. 1971 3;9(1):97–113. 5146491 10.1016/0028-3932(71)90067-4

[26] Löwe B, Kroenke K, Herzog W, Gräfe K. Measuring depression outcome with a brief self-report instrument: sensitivity to change of the Patient Health Questionnaire (PHQ-9). Journal of Affective Disorders. 2004 7 2004;81(1):61–6. 10.1016/S0165-0327(03)00198-8 15183601

[27] Cohen S, Kamarck T, Mermelstein R. A global measure of perceived stress. J Health Soc Behav. 1983 12;24(4):385–96. 6668417

[28] Cohen JD, Perlstein WM, Braver TS, Nystrom LE, Noll DC, Jonides J, et al Temporal dynamics of brain activation during a working memory task. Nature. 1997 4 10;386(6625):604–8. 10.1038/386604a0 9121583

[29] Gevins A, Cutillo B. Spatiotemporal dynamics of component processes in human working memory. Electroencephalogr Clin Neurophysiol. 1993 9;87(3):128–43. 7691540 10.1016/0013-4694(93)90119-g

[30] Miller KM, Price CC, Okun MS, Montijo H, Bowers D. Is the N-Back Task a Valid Neuropsychological Measure for Assessing Working Memory? Archives of Clinical Neuropsychology. 2009 11 01;24(7):711–7. 10.1093/arclin/acp063 19767297 PMC2770861

[31] Tzourio-Mazoyer N, Landeau B, Papathanassiou D, Crivello F, Etard O, Delcroix N, et al Automated Anatomical Labeling of Activations in SPM Using a Macroscopic Anatomical Parcellation of the MNI MRI Single-Subject Brain. Neuroimage. 2002 1;15(1):273–89. 10.1006/nimg.2001.0978 11771995

[32] Nichols T, Hayasaka S. Controlling the familywise error rate in functional neuroimaging: a comparative review. Stat Methods Med Res. 2003 Oct;12(5):419–46. 10.1191/0962280203sm341ra 14599004

[33] Schulze ET, Geary EK, Susmaras TM, Paliga JT, Maki PM, Little DM. Anatomical correlates of age-related working memory declines. J Aging Res. 2011;2011:606871 10.4061/2011/606871 22175019 PMC3228338

[34] Schmidt H, Jogia J, Fast K, Christodoulou T, Haldane M, Kumari V, et al No gender differences in brain activation during the N-back task: an fMRI study in healthy individuals. Hum Brain Mapp. 2009 11;30(11):3609–15. 10.1002/hbm.20783 19387979 PMC6870785

[35] Niendam TA, Laird AR, Ray KL, Dean YM, Glahn DC, Carter CS. Meta-analytic evidence for a superordinate cognitive control network subserving diverse executive functions. Cogn Affective Behav Neurosci. 2012;12(2):241–68. 10.3758/s13415-011-0083-5 PMC3660731 22282036

[36] Owen AM, McMillan KM, Laird AR, Bullmore E. N-back working memory paradigm: A meta-analysis of normative functional neuroimaging studies. Hum Brain Mapp. 2005;25(1):46–59. 10.1002/hbm.20131 15846822 PMC6871745

[37] Burton H, Videen TO, Raichle ME. Tactile-vibration-activated foci in insular and parietal-opercular cortex studied with positron emission tomography: mapping the second somatosensory area in humans. Somatosens Mot Res. 1993;10(3):297–308. 8237217 10.3109/08990229309028839

[38] Wacker E, Spitzer B, Lutzkendorf R, Bernarding J, Blankenburg F. Tactile motion and pattern processing assessed with high-field FMRI. PLoS One. 2011;6(9):e24860 10.1371/journal.pone.0024860 21949769 PMC3174219

[39] Spence C, Gallace A. Recent developments in the study of tactile attention. Can J Exp Psychol. 2007;61(3):196–207. 17974314 10.1037/cjep2007021

[40] Johansen-Berg H, Christensen V, Woolrich M, Matthews PM. Attention to touch modulates activity in both primary and secondary somatosensory areas. Neuroreport. 2000;11(6):1237–41. 10817599 10.1097/00001756-200004270-00019

[41] Rottschy C, Langner R, Dogan I, Reetz K, Laird AR, Schulz JB, et al Modelling neural correlates of working memory: A coordinate-based meta-analysis. Neuroimage. 2012 3;60(1):830–46. 10.1016/j.neuroimage.2011.11.050 22178808 PMC3288533

[42] Jaeggi SM, Buschkuehl M, Perrig WJ, Meier B. The concurrent validity of the N-back task as a working memory measure. Memory. 2010 05;18(4):394–412. 10.1080/09658211003702171 20408039

[43] Curtis CE, D'Esposito M. Persistent activity in the prefrontal cortex during working memory. Trends Cogn Sci (Regul Ed). 2003 9;7(9):415–23. 12963473 10.1016/s1364-6613(03)00197-9

[44] Vossel S, Geng JJ, Fink GR. Dorsal and ventral attention systems: distinct neural circuits but collaborative roles. Neuroscientist. 2014 4;20(2):150–9. 10.1177/1073858413494269 23835449 PMC4107817

[45] Corbetta M., Patel G., Shulman G.L. The Reorienting System of the Human Brain: From Environment to Theory of Mind. Neuron. 2008;58(3):306–24. 10.1016/j.neuron.2008.04.017 18466742 PMC2441869

[46] Singh-Curry V, Husain M. The functional role of the inferior parietal lobe in the dorsal and ventral stream dichotomy. Neuropsychologia. 2009 5;47(6):1434–48. 10.1016/j.neuropsychologia.2008.11.033 19138694 PMC2697316

[47] Corbetta M, Shulman GL. Human cortical mechanisms of visual attention during orienting and search. Philos Trans R Soc Lond B Biol Sci. 1998 8 29;353(1373):1353–62. 10.1098/rstb.1998.0289 9770228 PMC1692334

[48] Summers IR, Francis ST, Bowtell RW, McGlone FP, Clemence M. A functional-magnetic-resonance-imaging investigation of cortical activation from moving vibrotactile stimuli on the fingertip. J Acoust Soc Am. 2009 2;125(2):1033–9. 10.1121/1.3056399 19206877

[49] Burton H, Sinclair RJ, McLaren DG. Cortical network for vibrotactile attention: a fMRI study. Hum Brain Mapp. 2008 2;29(2):207–21. 10.1002/hbm.20384 17390318 PMC2593407

[50] Van Boven R.W., Ingeholm J.E., Beauchamp M.S., Bikle P.C., Ungerleider L.G. Tactile form and location processing in the human brain. Proc Natl Acad Sci U S A. 2005;102(35):12601–5. 10.1073/pnas.0505907102 16116098 PMC1188260

[51] Kim J, Muller KR, Chung YG, Chung SC, Park JY, Bulthoff HH, et al Distributed functions of detection and discrimination of vibrotactile stimuli in the hierarchical human somatosensory system. Front Hum Neurosci. 2015 1 21;8:1070 10.3389/fnhum.2014.01070 25653609 PMC4301016

[52] Martuzzi R, van der Zwaag W, Farthouat J, Gruetter R, Blanke O. Human finger somatotopy in areas 3b, 1, and 2: a 7T fMRI study using a natural stimulus. Hum Brain Mapp. 2014 1;35(1):213–26. 10.1002/hbm.22172 22965769 PMC6869627

[53] Eickhoff SB, Amunts K, Mohlberg H, Zilles K. The human parietal operculum. II. Stereotaxic maps and correlation with functional imaging results. Cereb Cortex. 2006 2;16(2):268–79. 10.1093/cercor/bhi106 15888606

[54] Eickhoff SB, Grefkes C, Zilles K, Fink GR. The somatotopic organization of cytoarchitectonic areas on the human parietal operculum. Cereb Cortex. 2007 8;17(8):1800–11. 10.1093/cercor/bhl090 17032710

[55] Malinen S, Schurmann M, Hlushchuk Y, Forss N, Hari R. Improved differentiation of tactile activations in human secondary somatosensory cortex and thalamus using cardiac-triggered fMRI. Exp Brain Res. 2006 9;174(2):297–303. 10.1007/s00221-006-0465-z 16676169

[56] Johansen-Berg H, Christensen V, Woolrich M, Matthews PM. Attention to touch modulates activity in both primary and secondary somatosensory areas. Neuroreport. 2000 4 27;11(6):1237–41. 10817599 10.1097/00001756-200004270-00019

[57] Staines WR, Graham SJ, Black SE, McIlroy WE. Task-relevant modulation of contralateral and ipsilateral primary somatosensory cortex and the role of a prefrontal-cortical sensory gating system. Neuroimage. 2002 1;15(1):190–9. 10.1006/nimg.2001.0953 11771988

[58] Fujiwara N, Imai M, Nagamine T, Mima T, Oga T, Takeshita K, et al Second somatosensory area (SII) plays a significant role in selective somatosensory attention. Brain Res Cogn Brain Res. 2002 11;14(3):389–97. 12421662 10.1016/s0926-6410(02)00141-6

[59] Schubert R, Ritter P, Wustenberg T, Preuschhof C, Curio G, Sommer W, et al Spatial attention related SEP amplitude modulations covary with BOLD signal in S1—a simultaneous EEG—fMRI study. Cereb Cortex. 2008 11;18(11):2686–700. 10.1093/cercor/bhn029 18372293

[60] Van Hulle L, Van Damme S, Spence C, Crombez G, Gallace A. Spatial attention modulates tactile change detection. Exp Brain Res. 2013 1;224(2):295–302. 10.1007/s00221-012-3311-5 23109085

[61] Hoechstetter K, Rupp A, Meinck HM, Weckesser D, Bornfleth H, Stippich C, et al Magnetic source imaging of tactile input shows task-independent attention effects in SII. Neuroreport. 2000 8 3;11(11):2461–5. 10943704 10.1097/00001756-200008030-00024

[62] Hartmann S, Missimer JH, Stoeckel C, Abela E, Shah J, Seitz RJ, et al Functional connectivity in tactile object discrimination: a principal component analysis of an event related fMRI-Study. PLoS One. 2008;3(12):e3831 10.1371/journal.pone.0003831 19048104 PMC2585476

[63] Zhang M, Mariola E, Stilla R, Stoesz M, Mao H, Hu X, et al Tactile discrimination of grating orientation: fMRI activation patterns. Hum Brain Mapp. 2005 8;25(4):370–7. 15852384 10.1002/hbm.20107 PMC6871710

[64] Shiffrin RM, Schneider W. Controlled and automatic human information processing: II. Perceptual learning, automatic attending and a general theory. Psychol Rev. 1977;84(2):127–90.

[65] Schneider W, Shiffrin RM. Controlled and automatic human information processing: I. Detection, search, and attention. Psychol Rev. 1977;84(1):1–66.

[66] Wiens S, Szychowska M, Nilsson ME. Visual Task Demands and the Auditory Mismatch Negativity: An Empirical Study and a Meta-Analysis. PLoS One. 2016 1 7;11(1):e0146567 10.1371/journal.pone.0146567 26741815 PMC4704804

[67] Chung Y.G., Han S.W., Kim H.-S., Chung S.-C., Park J.-Y., Wallraven C., et al Adaptation of cortical activity to sustained pressure stimulation on the fingertip. BMC Neurosci. 2015;16(1). 10.1186/s12868-015-0229-4 PMC4625848 26514637

